# Socioeconomic and physical health status changes after visual impairment in Korea using difference-in-difference estimations

**DOI:** 10.1038/s41598-020-79760-7

**Published:** 2021-03-08

**Authors:** Hyero Kim, Heejo Koo, Euna Han

**Affiliations:** College of Pharmacy, Yonsei Institute of Pharmaceutical Sciences, Yonsei University, 162-1 Songdo-Dong, Yeonsu-Gu, Incheon, South Korea

**Keywords:** Health care, Health care economics, Public health

## Abstract

This study analyzed the impact of visual impairment on socioeconomic and physical health status and its heterogeneity by severity of visual impairment. We used nationally representative cohort data based on Korean national health insurance claims (2002–2013), which were extracted for 11,030 persons (2206 visually impaired, 8824 control). This was restructured as monthly data for each person (person-month). Multivariate and ordered logistic regressions were conducted, and the pre-impairment status between the visually impaired and non-visually impaired people was adjusted by difference-in-difference (DiD) estimation. Focusing on medical aid (a public healthcare service assistance program for people who cannot afford health insurance premiums), the DiD estimate showed that the likelihood of receiving aid was higher among visually impaired compared with non-impaired people. Mildly and severely visually impaired people were more likely to be medical aid recipients than their counterparts. The severely visually impaired group was more likely to be unemployed. The visually impaired group were less likely to have no comorbidity. Our findings show that the socioeconomic and physical health status of visually impaired people is more likely to deteriorate than that of their non-visually impaired counterparts following onset of impairment.

## Introduction

Visual impairment is the third most common impairment globally^1^. Visual impairment was also ranked as the fourth most common impairment in South Korea, and approximately 253,000 people were registered as visually impaired in 2018 in the country^2^. The incidence rate of visual impairment increases rapidly in age groups over 50 years^3^, which implies that ageing and geriatric diseases are important determinants of visual impairment^3^. Because Korea is ageing faster than any other county, it is likely that visual impairment resulting from geriatric diseases will become a particularly significant public health concern in Korea^4^.

Visual impairment poses a considerable economic burden at the individual and family levels as well as to society overall because of increasing health care expenditure stemming from direct and indirect pathways: direct medical costs are incurred for hospitalization and medical services relating to diagnosis^5^ and indirect medical costs occur mainly for assistive care^6^. Visually impaired people may also experience economic and general health challenges at personal level and more broadly at the familial level with regard to in-house caregiving^6^. They are more likely to live in a low-income environment^3^ and experience other personal health burdens such as depression, loss of independence, and reduced quality of life, all of which are recognized as intangible costs of visual impairment^7–9^. Limitations in mobility and daily activities together with other health risks related to falls or injuries are also associated with the recurrent use of healthcare services, presenting challenges for chronic disease management^10–13^. Both medical and non-medical burdens of visual impairment were also more significant for those with progressive vision loss^12,14–16^. However, there are a few studies on how the incidence of visual impairment affects socioeconomic status^17^. Most previous studies on health of visually impaired people focused only on the hazard of visual impairment caused by specific eye conditions, such as glaucoma or cataracts^17,18^, and usually only included the elderly and rarely accounted for impairment severity.

The present study investigated the impact of visual impairment on socioeconomic and physical health status and its heterogeneity by severity of visual impairment. We used retrospective cohort data over the sample period (2002–2013) based on Korean National Health Insurance Service (NHIS) claims, which is nationally representative and enables real-time utilisation of healthcare data in South Korea. We strengthened the internal validity of the estimations by controlling for the pre-impairment status of the visually impaired person and constructing their counterparts (not visually impaired) through propensity score matching. Healthcare seeking behaviours or practices of the visually impaired need to be addressed to understand social and medical needs among the visually impaired more clearly^14^. This is particularly important as social capital for disability is not established as the same rate as economic growth in rapidly developing countries, such as South Korea. The results of this study will add global evidence on the burden of visual impairment for the individual.

## Results

The unemployment rate was 58.8% in the visually impaired group, which was slightly lower than the control group (at 59.07%). The proportions of 0 or 1 Charlson Comorbidity Index (CCI) score were higher in the control group; however, this was the opposite for higher CCI scores. More than half of both the visually impaired and control groups were dependents of the primary insured person in the household. The proportion of medical aid recipients was approximately 4% in the visually impaired group, whereas it was approximately 1.9% for the control group (Table 1).

**Table 1:** Summary statistics (unit: person-month).

	N (%), Mean ± SD	
	Visually impaired (N = 160,234)	Non visually impaired (N = 591,578)
**Dependent variables**		
*Income level*		
Medical aid	7594 (4.74)	13,172 (2.23)
Low income	50,998 (31.83)	164,835 (27.86)
Middle income	56,293 (35.13)	219,403 (37.09)
High income	45,349 (28.3)	194,168 (32.82)
*No work*		
Yes	94,219 (58.8)	349,429 (59.07)
No	66,015 (41.2)	242,149 (40.93)
*Comorbidity status*		
Charlson comorbidity index	0.7490 ± 1.1076	0.5500 ± 0.8925
Charlson comorbidity index = 0	91,461 (57.08)	375,688 (63.51)
Charlson comorbidity index = 1	37,083 (23.14)	140,482 (23.75)
Charlson comorbidity index = 2	19,139 (11.94)	51,183 (8.65)
Charlson comorbidity index ≥ 3	12,551 (7.83)	24,225 (4.09)
**Covariates**		
*Gender*		
Female (reference)	88,211 (55.05)	331,222 (55.99)
Male	72,023 (44.95)	260,356 (44.01)
*Age group*		
Young adults (reference)	11,441 (7.14)	37,226 (6.29)
Adults	68,418 (42.7)	245,294 (41.46)
Elderly	80,375 (50.16)	309,058 (52.24)
*Health insurance qualifications*		
Self-employed	38,823 (24.23)	137,405 (23.23)
Employee	20,475 (12.78)	93,200 (15.75)
Medical aid	6,717 (4.19)	11,544 (1.95)
Dependents (reference)	94,219 (58.81)	349,429 (59.07)

**Figure 1:**
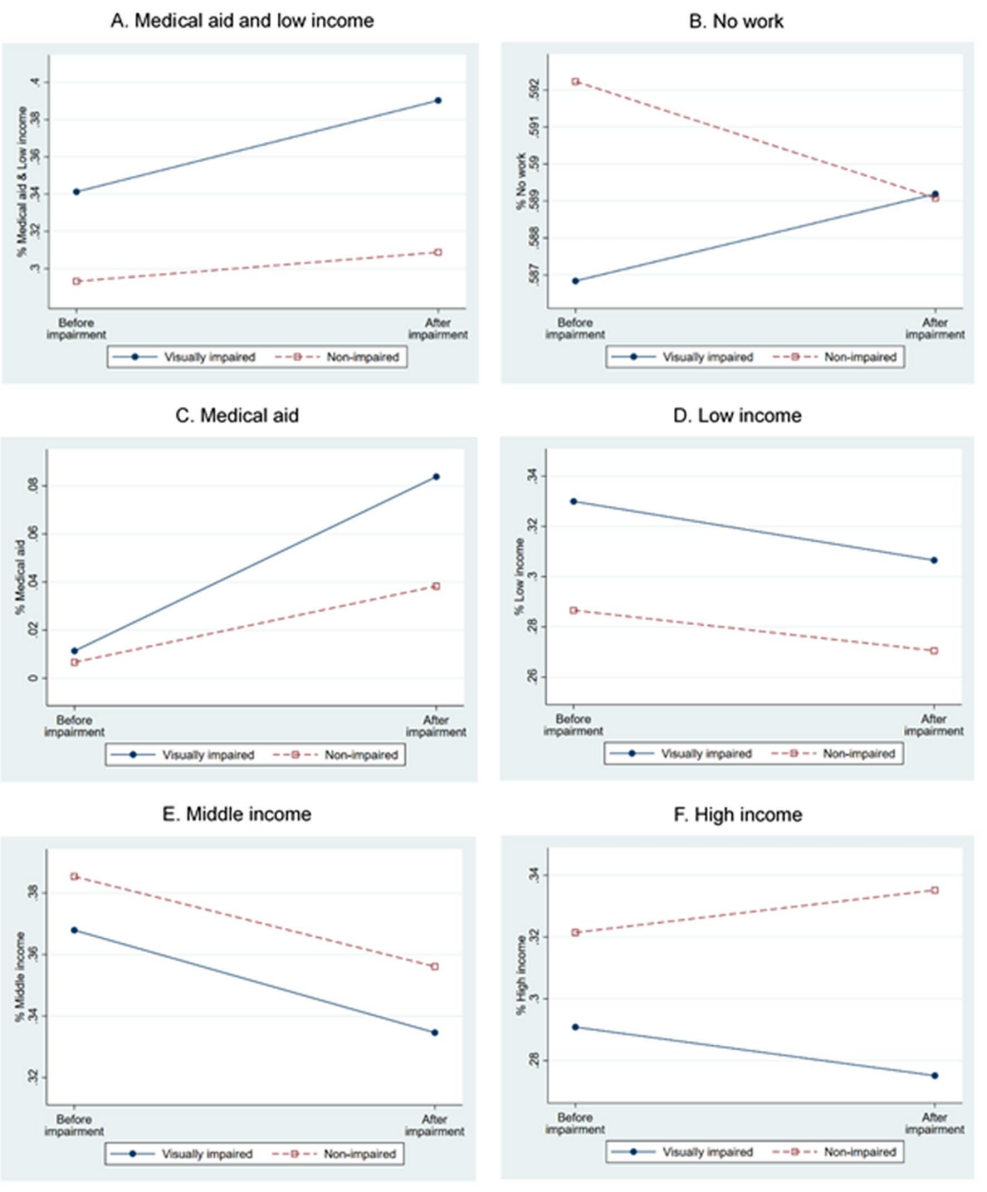
Proportion change of socioeconomic status by visual impairment. (**a**) Medical aid or low income; (**b**) No work; (**c**) Medical aid; (**d**) Low income; (**e**) Middle income; (**f**) High income. Each solid line represents for visually impaired and each dotted line represents for non-impaired. Each *X* axis denotes two time points, i.e., before and after visual impairment.

**Figure 2:**
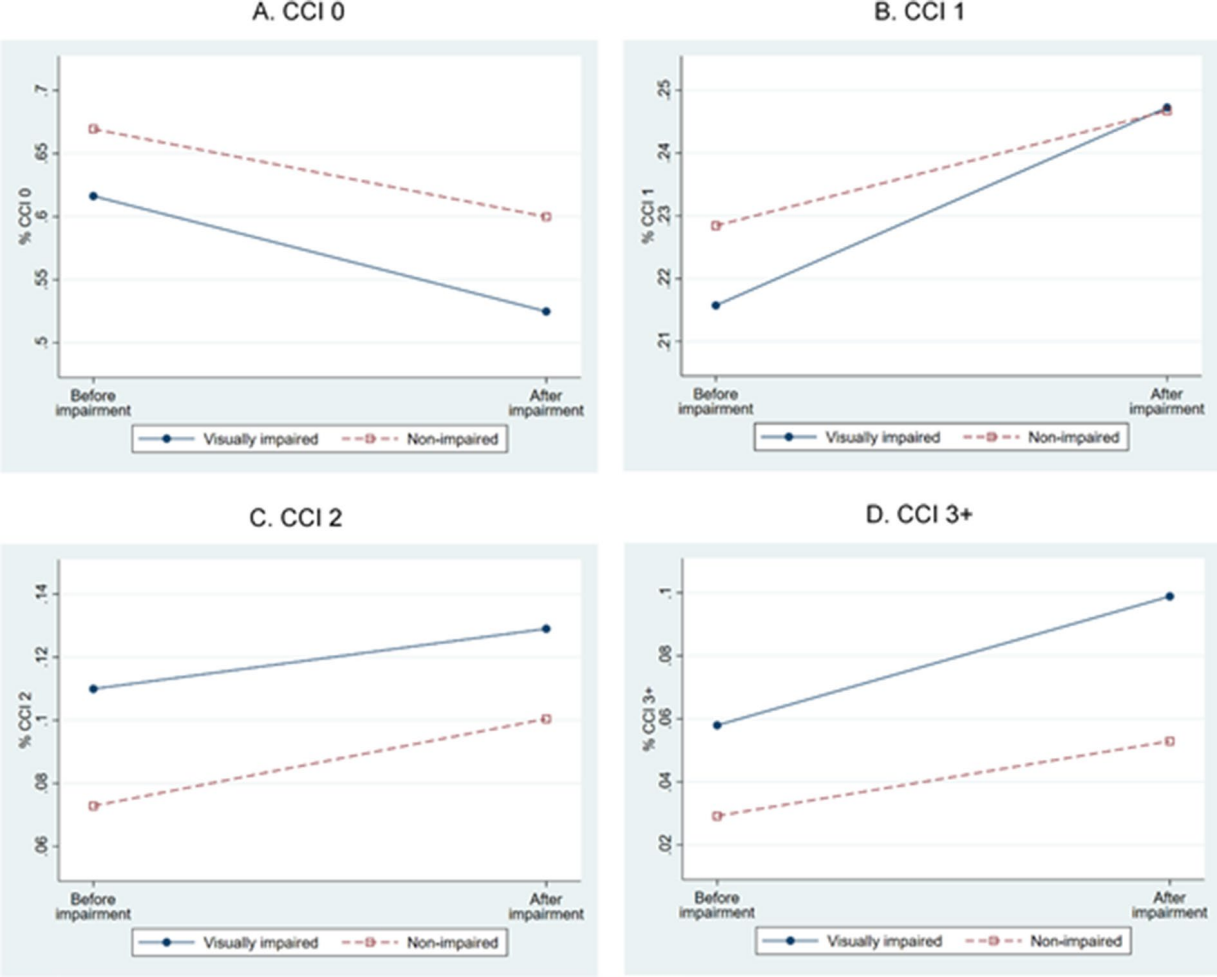
Proportion change of CCI by visual impairment.

Crude prevalence rates for socioeconomic status and the CCI group are presented in Figs. 1 and 2, respectively. The average proportions of medical aid and low income and medical aid only were higher in the visually impaired group than the control group at pre-impairment. Moreover, the proportion increased further post-impairment; the magnitude of the increase was larger in the visually impaired group than the control group for both variables. For the unemployment rate, the pre-impairment proportion was slightly lower in the visually impaired group than the control group, which was reversed post-impairment (Fig. 1). For the CCI group, the proportion of 0 CCI score decreased for both visually impaired and control groups, whereas that of all other CCI groups increased. The proportion of CCI score 1 was higher in the control group than the impairment group before the onset of visual impairment; however, the visually impaired group almost attained parity with the control group after visual impairment (Fig. 2).

The socioeconomic status and physical health status of visually impaired people are more likely to deteriorate after the onset of the impairment compared with non-visually impaired people. The visually impaired were more likely to be in the medical aid group than the non-impaired, even when adjusting the pre-impairment difference between the two groups (adjusted odds ratio (aOR) 1.0265, 95% confidence interval (CI) 1.0199–1.0331). Results also show that the visually impaired were more likely to be in medical aid or on a low income group than the non-impaired control group, even after adjusting the pre-impairment difference between the two groups (aOR 1.0147, 95% CI 1.0117–1.0177). However, no statistically significant results were estimated for the probability of no work at the 5% significance level. The visually impaired were more likely to be recipients of medical aid (aOR 1.0095, 95% CI 1.0083–1.0107), whereas they were less likely to be on a high income (aOR 0.9733, 95% CI 0.9708–0.9758). With regard to physical health, the difference-in-difference (DiD) estimates indicate that the visually impaired were less likely to have 0 CCI score (aOR 0.9737, 95% CI 0.9703–0.9771) and more likely to have a CCI score of 3 or more higher than the control group (aOR 1.0135, 95% CI 0.9703–0.9771) (Table 2).

**Table 2:** The estimated effect of visual impairment on socioeconomic and health status (N = 751,812).

Difference between person with and without visual impairment	Odds ratio (95% CI)			
	Medical aid and Low income	Medical aid	No work	
**From logistic regressions**				
Pre-impairment	1.1099 (1.0893, 1.1309)	1.0045 (1.0027, 1.0063)	1.0031 (0.9943, 1.0120)	
Post-impairment	1.1265 (1.1060, 1.1473)	1.0339 (1.0258, 1.0420)	1.0042 (0.9960, 1.0125)	
Difference-in-difference	1.0147 (1.0117, 1.0177)	1.0265 (1.0199, 1.0331)	1.0011 (0.9986, 1.0037)	

	Income group^b^			
	Medical aid	Low income	Middle income	High income
**From ordered logistic regressions**				
Pre-impairment	1.0215 (1.0157, 1.0273)	1.0547 (1.0428, 1.0667)	0.9988 (0.9978, 0.9998)	0.9292 (0.9143, 0.9444)
Post-impairment	1.0308 (1.0244, 1.0373)	1.0756 (1.0641, 1.0872)	0.9978 (0.9962, 0.9994)	0.9039(0.8899, 0.9181)
Difference-in-difference	1.0095 (1.0083, 1.0107)	1.0189 (1.0167, 1.0211)	0.9988 (0.9982, 0.9994)	0.9733 (0.9708, 0.9758)

	Charson comorbidity index group			
	Charson comorbidity index 0	Charson comorbidity index 1	Charson comorbidity index 2	Charson comorbidity index 3+
**From ordered logistic regressions**				
Pre-impairment	0.9474 (0.9337, 0.9612)	1.0247 (1.0183, 1.0311)	1.0166 (1.0122, 1.0210)	1.0133 (1.0095, 1.0171)
Post-impairment	0.9220 (0.9075, 0.9367)	1.0306 (1.0250, 1.0363)	1.0244 (1.0196, 1.0292)	1.0274 (1.0214, 1.0334)
Difference-in-Difference	0.9737 (0.9703, 0.9771)	1.0059 (1.0043, 1.0075)	1.0075 (1.0063, 1.0087)	1.0135 (1.0111, 1.0159)

^a^*p < 0.10, **p < 0.05, ***p < 0.01.

^b^Medical aid, Low income (1st to 4th deciles), Middle income (5th to 8th deciles), High income (9th to 10th deciles).

After dividing the visual impairment group into mild and severe groups, The DiD estimates were established, as presented in Table 3. When the pre-impairment difference between each impairment group and their counterparts was adjusted, the likelihood of being medical aid recipients or in a low-income group was higher for both mild and severe visual impairment groups (aOR 1.0140, 95% CI 1.0108–1.0172 and aOR 1.0130, 95% CI 1.0057–1.0204, respectively). For the probability of being medical aid recipients after adjusting the pre-impairment difference, the DiD estimates showed a higher likelihood for both mild and severe impairment groups (aOR 1.0258, 95% CI 1.0194–1.0322 and aOR 1.0551, 95% CI 1.0418–1.0686, respectively) than the control group. The DiD estimate for the likelihood of having no work was only statistically significant for the severely visually impaired group with the extent of the estimate being trivial (aOR 1.0069, 95% CI 1.0000–1.0138). With regard to income level, visually impaired individuals were less likely to be on a high income (aOR 0.9753, 95% CI 0.9726–0.9780 for the mild impairment group; aOR 0.9655, 95% CI 0.9585–0.9725 for the severe impairment group). Clear gradients in the penalty for CCI were estimated by the magnitude of the visual impairment: the mildly impaired group was less likely (aOR 0.9787, 95% CI 0.9751–0.9823) to have 0 CCI score and more likely to have a higher than 3 CCI score (aOR 1.0106, 95% CI 1.0082–1.0130), whereas the corresponding magnitudes were higher for the severe impairment group (aOR 0.9450, 95% CI 0.9371–0.9530; aOR 1.0446, 95% CI 1.0350–1.0542).

**Table 3:** The estimated effect of visual impairment on socioeconomic and health status by severity of impairment (N = 751,812).

Difference between person with and without visual impairment	Odds ratio (95% CI):			
	Medical aid and low income	Medical aid	No work	
**From logistic regressions**				
*Mild visual impairment*				
Pre-impairment	1.1084 (1.0868, 1.1304)	1.0054 (1.0034, 1.0074)	0.9998 (0.9907. 1.0090)	
Post-impairment	1.1249(1.1036, 1.1466)	1.0339 (1.0256, 1.0422)	1.0000 (0.9914, 1.0086)	
Difference-in-difference	1.014 (1.0108, 1.0172)	1.0258 (1.0194, 1.0322)	1.0002 (0.9975, 1.0029)	
*Severe visual impairment*				
Pre-impairment	1.1128 (1.0594, 1.1689)	1.0081 (1.0038, 1.0125)	1.0288 (1.0066, 1.0515)	
Post-impairment	1.1275 (1.0746, 1.1830)	1.0665 (1.0498, 1.0835)	1.0357 (1.0169, 1.0549)	
Difference-in-difference	1.0130 (1.0057, 1.0204)	1.0551 (1.0418, 1.0686)	1.0069 (1.0000, 1.0138)	

	Income group^b^			
	Medical aid	Low income	Middle income	High income
**From ordered logistic regressions**				
*Mild visual impairment*				
Pre-impairment	1.0208 (1.0146, 1.0270)	1.0526 (1.0401, 1.0652)	0.9986 (0.9976, 0.9996)	0.9319 (0.9162, 0.9479)
Post-impairment	1.0294 (1.0288, 1.0361)	1.0721 (1.0600, 1.0843)	0.9974 (0.9958, 0.9990)	0.9085 (0.8937, 0.9235)
Difference-in-difference	1.0089 (1.0077, 1.0101)	1.0177 (1.0155, 1.0199)	0.9986 (0.9980, 0.9992)	0.9753 (0.9726, 0.9780)
*Severe visual impairment*				
Pre-impairment	1.0320 (1.0125, 1.0519)	1.0644 (1.0384, 1.0911)	0.9957 (0.9906, 1.0008)	0.9144 (0.8796, 0.9506)
Post-impairment	1.0512 (1.0285, 1.0744)	1.0900 (1.0665, 1.1141)	0.9894 (0.9805, 0.9983)	0.8821 (0.8523, 0.9130)
Difference-in-difference	1.0192 (1.0150, 1.0182)	1.0232 (1.0182, 1.0282)	0.9931 (0.9888, 0.9974)	0.9655 (0.9585, 0.9725)

	Charson comorbidity index group			
	Charson comorbidity index 0	Charson comorbidity index 1	Charson comorbidity index 2	Charson comorbidity index 3+
**From ordered logistic regressions**				
*Mild visual impairment*				
Pre-impairment	0.9578 (0.9437, 0.9721)	1.0197 (1.0129, 1.0265)	1.0133 (1.0087, 1.0179)	1.0106 (1.0067, 1.0146)
Post-impairment	0.9370 (0.9216, 0.9527)	1.0245 (1.0185, 1.0305)	1.0196 (1.0146, 1.0246)	1.0216 (1.0154, 1.0278)
Difference-in-difference	0.9787 (0.9751, 0.9823)	1.0049 (1.0033, 1.0065)	1.0062 (1.0050, 1.0074)	1.0106 (1.0082, 1.0130)
*Severe visual impairment*				
Pre-impairment	0.8623 (0.8258, 0.9004)	1.0618 (1.0466, 1.0773)	1.0456* (1.0324, 1.0590)	1.0446 (1.0278, 1.0617)
Post-impairment	0.8143 (0.7794, 0.8508)	1.0562 (1.0502, 1.0622)	1.0641 (1.0494, 1.0790)	1.0926 (1.0657, 1.1202)
Difference-in-difference	0.9450 (0.9371, 0.9530)	0.9957 (0.9866, 1.0049)	1.0173 (1.0145, 1.0201)	1.0446 (1.0350, 1.0542)

^a^*p* < 0.10, **p < 0.05, ***p < 0.01.

^b^Medical aid, Low income (1st to 4th deciles), Middle income (5th to 8th deciles), High income (9th to 10th deciles).

## Discussion

Our findings show that the socioeconomic status of the visually impaired (compared with the control group) worsened after the onset of visual impairment, showing an increase in the likelihood of being on a low-income and unemployed. Physical health also deteriorated. Moreover, those changes were overall more significant for the severe visual impairment group than for the mild impairment group.

This study has demonstrated that employed people and medical aid recipients constitute 12.78% and 4.19% of the visually impaired population in Korea, respectively, while the corresponding proportions of the Korean population in total in 2018 were 33.26% and 2.82%, respectively^19^. Korea operates a social security system, which includes disability pensions for people with severe disabilities, disability allowances for people with mild disabilities, and an obligatory employment system for disabled people to secure income and employment^20–22^. Despite these public efforts, people with visual impairments seem to be more vulnerable to poverty by being excluded from the labour market or receiving lower incomes than non-disabled people even where they do participate^23^.

Significant correlations between visual impairment and income level changes were repeatedly observed in all estimations in the present study, which is notable given that income level is a key factor in determining socioeconomic status. However, to the best of our knowledge, few studies have focused on the causality between visual impairment and changes in individual income level. This is because collecting data on income level, a highly sensitive subject for individuals in Korea, is difficult, and could have a negative effect on study participants^24^. The current study overcame such a problem by using health insurance premiums as an objective proxy for income level.

Our estimations show that the incremental likelihood of entering the medical aid group was greater among those who are severely visually impaired, whereas the likelihood for entering medical aid or a low-income group was slightly higher in the mild visual impairment group. These results indicate that individuals with a mild visual impairment were likely to be in the low-income group, which has relatively fewer benefits than the medical aid group. These results may imply that people with mild visual impairment (a much larger group than those with severe impairment)^25^ were vulnerable for health and welfare because most current benefits for people with disabilities focus on those with severe impairment^4,14^. The results for unemployment were statistically significant when the visually impaired group was divided into subgroups according to severity; that is, the extent of unemployment was only affected by severe visual impairment but not mild visual impairment. Results for correlations between employment status and visual impairment are controversial^25–27^. Therefore, future studies need to assess unemployment separately according to voluntary unemployment (such as retirement) compared with non-voluntary or enforced unemployment.

Our results also show that visually impaired people are vulnerable in terms of physical healthcare, which is also consistent with previous studies^6,15,28^. The negative effect of visual impairment was greater for higher CCI groups. Furthermore, individuals with severe visual impairment demonstrated a much greater average likelihood of having higher CCI than the mildly impaired group. This might imply that there are unmet medical needs among visually impaired people, particularly among those who are severely impaired, due to limited accessibility to medical institutions and health-related information^29^. Previous studies reported that visual impairment can also substantially disrupt individuals' daily lives^30,31^, activities of daily living (ADL), and instrumental ADL^32,33^. Furthermore, people with visual impairment demonstrated deterioration in self-assessed health and poor mental health^34,35^. It was also found that visually impaired people who also have diabetes, hypertension, or depression experienced greater difficulties with functioning and socialising than non-visually impaired people with the same conditions or those with neither visual impairment nor chronic diseases^28^. Visually impaired people may also experience reduced (or a lack of) basic healthy behaviours (such as physical activities and a balanced diet) due to their disability^36^, and are less engaged in physical activity due to both their visual impairment and obesity^24^. Understanding common comorbid conditions and lifestyle in relation to severity or duration of visual impairment would improve the overall health status of visually impaired people.

The effect of gender difference on the burden of visual impairment is well discussed in the previous literature. Variations by gender in terms of the causes of visual impairment indicated that for women, cataract, uncorrected refractive error, and diabetic retinopathy were more common visual impairments, while for men glaucoma and corneal opacity were more common. Age-related macular degeneration demonstrated no difference in attribution to visual impairment by gender^37^. Females are known to have higher rates of blindness globally^38^ even when controlling for socioeconomic status^39^. Unequal access to eye care services according to socioeconomic status (such as income and education levels) and by socio-cultural status (for example by gender) was considered a factor for visually impaired people, particularly in low income countries^38,40^. At the same time, gender difference associated with visual impairment and health status remains controversial^41,42^. Therefore, any potential discrepancy according to the effects of gender and the economic and health burdens of visually impaired people should be addressed in future research.

In our study, data are drawn from a representative sample of the whole Korean population; thus, the estimated differences between the visually impaired and the non-visually impaired control group can be accepted as real world based and externally valid. External validity is established given that people with disabilities in Korea are entitled to targeted assistance from the social security system and adjusted premiums for health insurance. Therefore, potential selection for access to healthcare services due to insurance premium is less of concern in our data^43^. Our study also demonstrates also strength in identifying visual impairment and extracting pre- and post-impairment information for relatively moderate duration and control for the pre-impairment difference between the visually impaired and non-impaired control group. This helps to ensure the internal validity of the results. At the same time, our data were drawn from the NHIS claims data, which were originally collected for claiming reimbursement for medical services and medication costs. Therefore, information not related to reimbursement claims (such as individual education level, occupation, marital status, or living arrangement) was lacking. For example, occupational status may be related to qualitative aspects of visual impairment, such as the cause of the impairment^44^. Despite these caveats, using panel data over 12 years together with a DiD model, it was possible to infer causality between the onset of visual impairment and changes in socioeconomic status or physical health, which were not addressed in previous cross-sectional studies.

Finally, negative changes for income level, employment status, and physical health were identified for visually impaired people after the onset of the impairment, compared with their non-visually impaired counterparts. The extent of changes were greater for those with a severe impairment compared with those with a mild impairment.

## Methods

### Data and study subjects

We used the Sample Cohort database from the Korean National Health Insurance Service (NHIS) claims data, which is public data released by the NHIS. From this, approximately 1 million NHIS beneficiaries were randomly selected in 2002 and followed up until 2013. The NHIS is the only public health insurer in Korea, to which all Koreans are registered as compulsory beneficiaries together with healthcare providers as mandatory insurance takers. According to nationwide statistics for 2018, 97.2% of the total population in Korea were covered by the NHIS, and the remaining 2.8% were supported by the Medical Aid Program (a public assistance program for healthcare services for qualified low-income people who cannot afford insurance premiums). The database also contains details on enrolees' income level represented by insurance premium level in deciles and occupational status (self-employed vs. employees), which were collected to determine recipients' qualification for the NHIS.

Disabled people can acquire various medical and rehabilitation benefits once they are formally registered with the local government, and registration requires a physician’s accurate assessment of their disability type and magnitude^45^. The NHIS system is linked with the registration system, having information on type, severity, and registration date of the registered disabled person. Approximately 94.1% of the total number of people with disabilities and 94.7% of visually impaired people were registered with the local government^19^.

**Figure 3:**
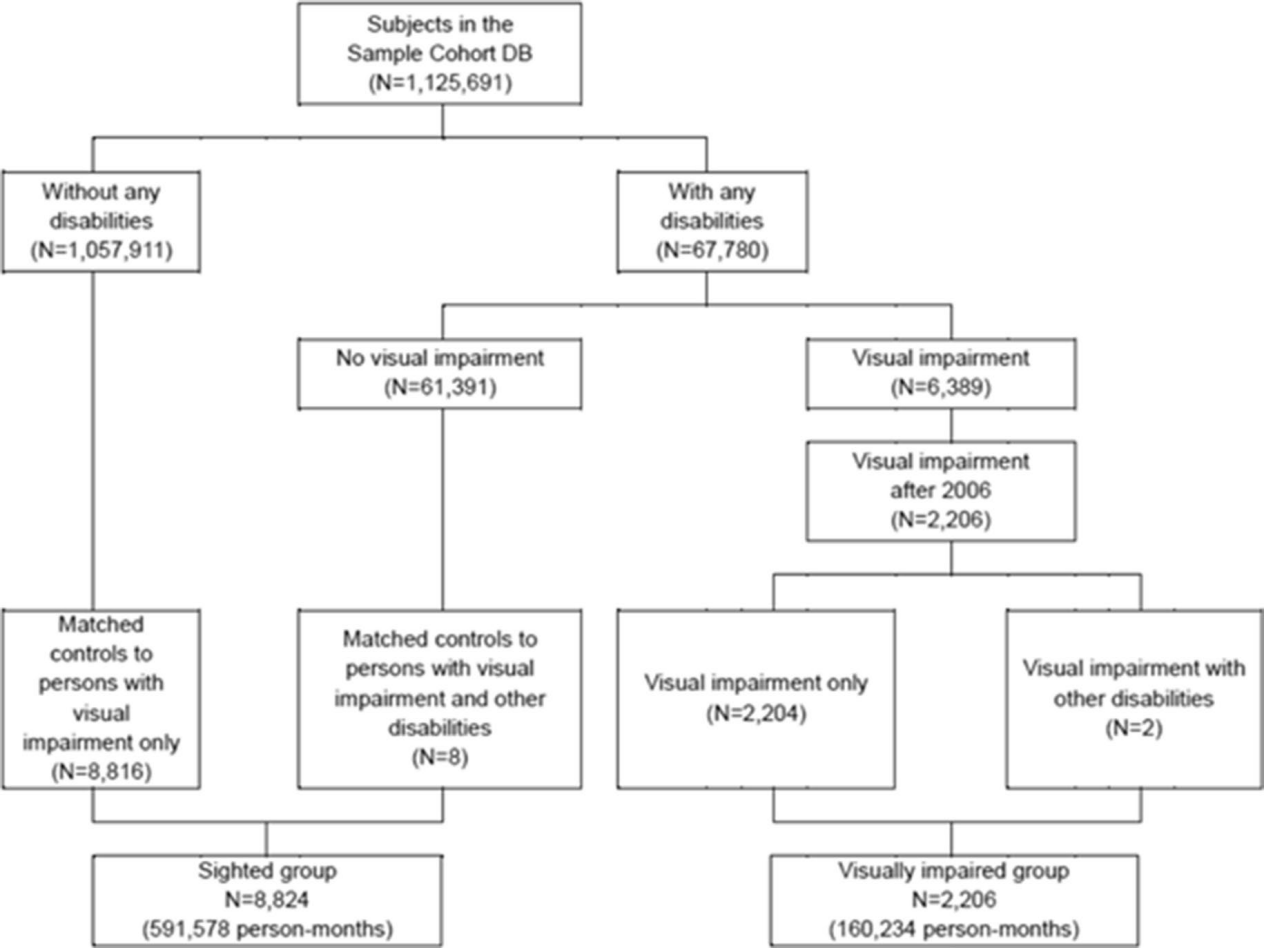
Sample selection process.

Figure 3 shows the process of sample selection for the present study. Of the 1,125,691 subjects, 67,780 people were identified with disabilities, and in particular, 6389 people had a visual impairment. We only included 2206 people with a visual impairment after 2006 to identify pre-impairment health and socioeconomic status for 2002–2005. The control group included those without any disabilities or those with disabilities other than visual impairment as respective counterparts of the visual impairment only group and the visual impairment with other disabilities group. We matched the visual impairment group and control group using a ratio of 1:4 with a propensity score matching method including age in years, gender, and residential region as the matching variables. Thus, 8824 people were included in the control group. NHIS claims data were extracted for the final sample (2206 visually impaired people and 8824 controls) and restructured as a monthly record for each sample person, generating 751,812 person-months (N = 160,234 for the visual impairment group; N = 591,578 for the control group).

### Variables

We used four indicators for socioeconomic status and one for physical health as dependent variables. For socioeconomic status, insurance premium was used as a proxy for income level. The four dependent variables for socioeconomic status were (1) a binary indicator for medical aid receipt; (2) a binary indicator for medical aid receipt or on low income; (3) a binary indicator for unemployment; and (4) ordinal categories of income, which were: very low (i.e., medical aid receipt), low (1st to 4th deciles of insurance premium), middle (5th to 8th deciles), and high income (9th to 10th deciles). For physical health, the CCI was used as a dependent variable, which was categorised as 0, 1, 2, and 3 or higher. The CCI has been widely used as an indicator for composite health status and is calculated as a weighted summed count for 19 diseases, where weights (points) are as follows: 1 = myocardial infarction, congestive heart failure, peripheral vascular disease, dementia, cerebrovascular disease, chronic lung disease, connective tissue disease, ulcer, chronic liver disease, diabetes; 2 = hemiplegia, moderate or severe kidney disease, diabetes with end organ damage, tumor, leukemia, lymphoma;, 3 = moderate or severe liver disease; or 6 = malignant tumor, metastasis, AIDS. These are applied according to the risk of death associated with each disease^46^. The CCI was scored using the International Classification of Diseases (ICD-10) codes (see “Appendix”).

The key independent variables were categorized as the presence and severity of visual impairment. Although visual impairment consists of visual acuity impairment and visual field impairment, we did not use this categorization because visual acuity impairment accounts for the majority of visual impairment cases. Diagnosis from an ophthalmologist is necessary to register a person with visual impairment to the local government. The *Enforcement Decree of the Act on Welfare of Persons with Disabilities* determines disability level cited in the registration as follows: Grade 1 (best-corrected visual acuity (BCVA) ≤ 0.02 in the better eye); Grade 2 (BCVA ≤ 0.04 in the better eye); Grade 3 (BCVA ≤ 0.06 in the better eye); Grade 4 (BCVA ≤ 0.1 in the better eye); Grade 5 (BCVA ≤ 0.2 in the better eye); and Grade 6 (BCVA ≤ 0.02 in the worse eye)^47^. The present study categorized Grades 1–3 as severe disability and Grades 4–6 as mild disability.

Moreover, we segmented the observed time as pre-impairment, baseline, and post-impairment. The baseline was defined as 1 year before the subject's date of registration as being visually impaired, considering an administrative lag of at least six months to actual registration after the clinical onset of visual impairment; the times before and after the baseline were respectively defined as pre- and post-impairment^48^.

We controlled gender and age group as covariates in all estimations. Age was organized into three categories: young adults (20–39 years, reference), adults (40–65 years), and elderly (> 65 years). We also controlled for 19 comorbid conditions as a series of dummy indicators to correct subjects' health status when estimating for socioeconomic status. Health insurance qualification types were controlled for in estimating physical health using a series of dummy indicators. These were self-employed, employees, and medical aid recipients, with non-employed and dependents of the primary insured as the reference group.

### Estimations

We used multivariate logit regressions for binary dependent variables and multivariate ordered-logit regressions for ordinal dependent variables. Unobserved characteristics at the individual level were controlled by using a mixed-effects model. The observation unit was person-month. We also further categorized visual impairment into mild and severe impairment groups and replicated all estimations.

We used DiD models to estimate the impact of visual impairment on both socioeconomic and health status compared with the control group, also controlling for pre-impairment differences between the two groups. The DiD estimation allows evaluation of the average treatment effect on the dependent variables of the treatment group (the visually impaired) compared with the control group (the sighted) by controlling background changes in outcomes that occur with time^48^.

The first estimation equation for this study is as follows:

1 $$ Y_{it} = \beta_{0} + \beta_{1} VI_{it} + \beta_{2} Post_{it} + \beta_{12} (VI_{it} \times Post_{it} ) + \beta_{3} X_{it} + \mu_{i} + \varepsilon_{it} $$

where subscripts *i* and *t* indicate individual and month, respectively. Term *Y* represents a series of dependent variables, while *VI* and *Post* are dummy indicators representing 1 if visually impaired and post-impairment, respectively, and 0 otherwise. Term *X* is a vector of covariates, and term μ denotes individual-level permanent unobserved characteristics. Term ε denotes the time-varying error component, which was assumed to be independently and identically distributed. Coefficient β in Eq. (1) represents the parameters to be estimated, and particularly, β_12_ represents the parameter for the DiD estimate.

The DiD estimates for visual impairment by severity is similarly derived as in Eq. (2). Terms *VI*^*mild*^ and *VI*^*sev*^ are dummy indicators representing 1 if the visual impairment is either mild or severe, respectively, and 0 otherwise. All other variables are the same as those in Eq. (1).

2 $$ Y_{it} = \beta_{0} + \beta_{1} VI_{it}^{mild} + \beta_{2} VI_{it}^{sev} + \beta_{3} Post + \beta_{13} (VI_{it}^{mild} \times Post) + \beta_{23} (VI_{it}^{sev} \times Post) + \beta_{4} X_{it} + \mu_{i} + \varepsilon_{it} $$

We inferred the interaction effect of visual impairment and post-impairment as the DiD estimate. The interaction effects are not constant; rather, they are conditional on the independent variables in nonlinear models, such as logit regression or ordered-logit regressions^49^. Hence, we reported the odds ratio of the DiD estimate. The extent of the DiD estimate was presented as the marginal effect, which is the difference in the adjusted probability for a given dependent variable. Thus, the DiD estimate is the average difference of the average difference between the visually impaired and control groups and the average difference between post- and pre-impairments.

We conducted all analyses using Stata 15.0 (StataCorp, College Station, TX, USA). This study was approved by the Yonsei institutional review board (7001988-201704-h-175-01E). All information in the Sample Cohort DB was provided after de-identification by the NHIS.

## Appendix: Charlson comorbidity index calculation

**Table:**

Comorbidity	ICD-10 code	Weight
Myocardial infarction	I21.x, I22.x, I25.2	1
Congestive heart failure	I09.94), I11.0, I13.0, I13.2, I25.5, I42.x, I43.x, I50.x	1
Peripheral vascular disease	I70.x, I71.x, I73.1, I73.8, I73.9, I77.1, I79.0, I79.2, K55.1, K55.8, K55.9, Z95.8, Z95.9	1
Cerebrovascular disease	I60.x, I61.x, I62.x, I63.x, I64.x, I65.x, I66.x, I67.x, I68. x 5), I69.x	1
Dementia	F00.x, F01.x, F02.x, F03.x, F05.1, G30.x, G31.1	1
Chronic lung disease	I27.8, I27.9, J40.x, J41.x, J42.x, J43.x, J44.x, J45.x, J46.x, J47.x, J60.x, J61.x, J62.x, J63.x, J64.x, J65.x, J66.x, J67.x, J68.4, J70.1, J70.3	1
Connective tissue disease	M05.x, M06.x, M32.x, M33.x, M34.x, M31.5, M35.1, M35.3, M36.0	1
Peptic ulcer	K25.x, K26.x, K27.x, K28.x	1
Mild liver disease	B18.x, K73.x, K74.x, K70.0, K70.1, K70.2, K70.3, K70.9, K71.3, K71.4, K71.5, K71.7, K76.0, K76.2, K76.3, K76.4, K76.8, K76.9, Z94.4	1
Diabetes without complications	E10, E11, E12, E13, E14, E10.0, E10.1, E10.6, E10.8, E10.9, E11.0, E11.1, E11.6, E11.8, E11.9, E12.0, E12.1, E12.6, E12.8, E12.9, E13.0, E13.1, E13.6, E13.8, E13.9, E14.0, E14.1, E14.6, E14.8, E14.9	1
Diabetes with Retinopathy, neurosis, kidney disease	E10.2, E10.3, E10.4, E10.5, E10.7, E11.2, E11.3, E11.4, E11.5, E11.7, E12.2, E12.3, E12.4, E12.5, E12.7, E13.2, E13.3, E13.4, E13.5, E13.7, E14.2, E14.3, E14.4, E14.5, E14.7	2
Hemiplegia	G81.x, G82.x, G04.1, G11.4, G80.1, G80.2, G83.0, G83.1, G83.2, G83.3, G83.4, G83.9	2
Severe abnormal kidney diseases	I12.0, I13.1, N03.2–N03.7, N05.2–N05.7, N18.x, N19.x, N25.0, Z49.0–Z49.2, Z94.0, Z99.2	2
Non-metastatic solid cancer, leukemia, lymphoma, multiple myeloma	C00.x–C26.x, C30.x–C34.x, C37.x–C41.x, C43.x, C45.x–C58.x, C60.x–C76.x, C81.x–C85.x, C88.x, C90.x–C97.x,	2
Severe abnormal liver disease	I85.0, I85.9, I86.4, I98.2, K70.4, K71.1, K72.1, K72.9, K76.5, K76.6, K76.7	3
Metastatic solid cancer	C77.x, C78.x, C79.x, C80.x	6
AIDS	B20.x, B21.x, B22.x, B24.x	6
